# The role of Plant Growth Promoting Bacteria in improving nitrogen use efficiency for sustainable crop production: a focus on wheat

**DOI:** 10.3934/microbiol.2017.3.413

**Published:** 2017-06-07

**Authors:** Nilde Antonella Di Benedetto, Maria Rosaria Corbo, Daniela Campaniello, Mariagrazia Pia Cataldi, Antonio Bevilacqua, Milena Sinigaglia, Zina Flagella

**Affiliations:** 1 Laboratory of Nutritional and Healthy Quality of Herbaceous Crop, Department of the Science of Agriculture, Food and Environment (SAFE) University of Foggia, Foggia, Italy; 2 Laboratory of Predictive Microbiology, Department of the Science of Agriculture, Food and Environment (SAFE), University of Foggia, Foggia, Italy

**Keywords:** PGPB, plant-bacteria interaction, rhizosphere, N-uptake, wheat, NUE

## Abstract

Due to the increase in both human population growth and environmental pressure, it is necessary to raise agricultural productivity without enhancing environmental footprint. Within this context, soil inoculation with PGPB (Plant Growth Promoting Bacteria) may be considered a promising tool of integrated management systems. In particular, PGPB may improve plant growth either directly, by facilitating resource use or modulating plant hormone levels, or indirectly by decreasing the inhibitory effects of various pathogenic agents. PGPB comprise different functional and taxonomic groups of bacteria like *Pseudomonas, Bacillus, Rhizobium* and others. Their ability to either mobilize mineral or organic bound nutrients from the pedosphere or to fix atmospheric N_2_ and make it available to the plants, is a crucial feature in their application. In literature some data are available on the use of commercial PGPB, while less efforts have been made on the study of the effect of autochthonous PGPB isolated from soils on sustainability of cropping systems; thus a literature survey on these aspects was carried out with special focus on wheat, a staple food for a large part of world population. In particular, the main topic of this review is the potential of PGPB to enhance use efficiency of agro-environmental resources focusing on the interaction PGPB-wheat for improving nitrogen use efficiency.

## 1. Introduction

The Food and Agriculture Organization of the United Nations (FAO) reported a projection of world population of 9.1 billion in 2050. The demands for major grain crops, such as wheat, are projected to 70% increase by 2050 [1] mainly through the increasing in crop intensity. In the past decades, agricultural practices aimed at maximizing yields mainly by increasing fertilization, without considering the socio-economic and ecological consequences [2],[3]. Indeed it would be of interest to ensure food production using sustainable technologies that reduce environmental impacts including ecosystem degradation and high greenhouse gas emissions. Sustainable intensification was defined as “maximization of primary production per unit area without compromising the ability of the system to sustain its productive capacity” [4]. The issue of primary production sustainability is more acute for wheat, which is the main cereal crop used for human consumption in many areas worldwide, it provides 50 percent of humanity's dietary energy supply with corn and rice [5]. Also durum wheat (*Triticum turgidum* L. subsp *durum*), a crop well adapted to Mediterranean basin, is a staple food for a part of world population being mainly used for pasta production [6].

Among fertilizers, nitrogen is the nutrient that is most susceptible to loss and its availability is affected by soil type, tillage, N-source, crop rotation and precipitation [7]. Moreover, its recovery by the crop is usually less than 50% of the applied amount [8]. However, present concerns about crop and environmental sustainability are putting added emphasis on increasing the nitrogen use efficiency (NUE) of crops. Improving NUE is among the main targets of crop research for Mediterranean environments [8].

Researchers, farmers, agricultural policy are focusing their attention towards potential innovative biotechnological solutions with lower environmental impact. Green biotechnologies have been proposed as new strategies for the management of the crops by improving the nutrient uptake efficiency, controlling biotic adversity, reducing the use of fertilizers, etc [9],[10]. It is known that some microorganisms (called Plant Growth Promoting Bacteria, PGPB) are able to influence biological nitrogen fixation, solubilize phosphate, produce phyto-hormones and other molecules, favor positive mycorrhizal-plant interactions and defend the plants from pathogenic bacteria.

In particular, soil inoculation with PGPB is a promising tool of integrated management systems to increase the efficiency of plants' use of nutrients (from either soil or fertilizers) through microbial technology and the sustainability of the cropping systems. PGPB are around/on the root surface and comprise different genera, like *Azospirillum, Azotobacter, Nitrobacter*, largely studied but few proficient to colonize root, and other genera such as *Bacillus, Pseudomonas, Bradyrhizobium, Acinetobacter, Klebsiella, Mesorhizobium, Rhizobium*, etc. that are proficient to colonize the root surface, survive and compete with other microbiota [11].

After an initial overview on environmental problems due to the crop intensification, this study reports (i) the use of PGPB to improve the sustainability of cropping systems; (ii) how PGPB interact with plants (in particular with wheat) for the improvement of resources uptake efficiency; (iii) a focus on NUE in wheat.

## 2. Greenhouse Gas (GHG) Emissions from Agricultural System

The annual demand for major grain crops, such as wheat, will need to rise to about 3 billion tons from 2.1 billion today [4],[12]. In opposition with the rapid increase of the world population, the rate of growth in agricultural production is expected to decrease as a consequence of climate changes. In particular, FAO's data show that annual crop production is expected to fall to 1.5% between now and 2030 and further to 0.9% between 2030 and 2050 [4].

Climate models predict a mean increase in temperature from 1.0 to 3.7 °C with an increase in frequency of heat waves by the end of 21^st^ century. Similarly, for rainfall patterns longer drought periods are predicted alternating with heavy rainfall, which will lead to flash floods events. In Europe, climate change is considered the main reason for decreasing yield growth rate in wheat. In particular, summer precipitations are predicted to decrease and heat waves will become more common and severe, with a negative impact on crop productivity [12],[13].

According to FAO the 90% of the growth in crop production will come from the agricultural intensification in particular from higher yields and increased cropping intensity. The remaining 10% will come from expanding arable land.

The agricultural intensification needed to increase crop production and food security, is linked to an increase in greenhouse gas (GHG) emissions. GHGs absorb infrared radiation in the atmosphere, trapping heat and warming the surface of the Earth. FAO's data show that GHGs emissions from agriculture including all the emissions produced in the different agricultural sectors (enteric fermentation, manure management, rice cultivation, synthetic fertilizers, manure applied to soils, manure left on pastures, crop residues, cultivation of organic soils, burning of crop residues, burning of savanna, energy use) have been estimated at 10% of total global emissions [14]. The main GHGs associated with agriculture are carbon dioxide (CO_2_), methane (CH_4_) and nitrous oxide (N_2_O) and the largest source of GHGs emissions within agriculture is enteric fermentation in ruminants, which is a major source of CH_4_ produced and released by livestock during ruminant digestion. In terms of the magnitude of emissions, it accounts for 40% of the whole agricultural GHGs emissions and is followed by the manure left on pasture (16%), the use of synthetic fertilizers (13%) and the rice cultivation (10%) [14].

**Figure 1.**
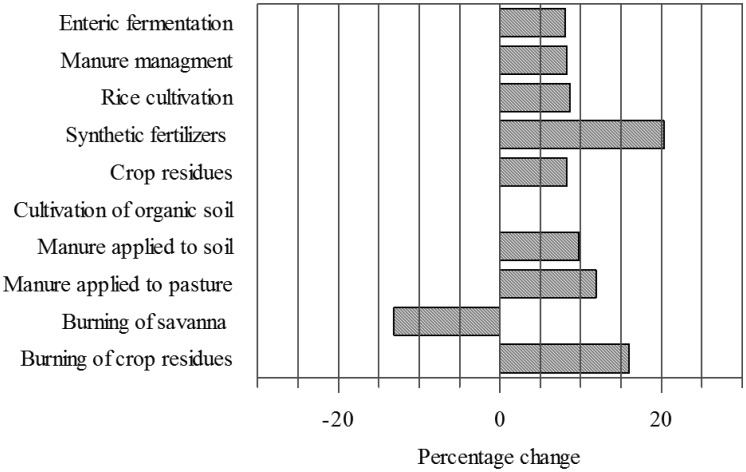
Percentage change in greenhouse gas emissions by agricultural source from 2004 to 2014 (data recovered from FAOSTAT [14] and modelled by the authors).

However while emissions from ruminant fermentation have increased by 8% between 2004 and 2014, emissions generated during the application of synthetic fertilizers, have increased by 20% since 2004 representing the fastest growing emissions source in agriculture (Figure 1).

In particular, world total annual emissions from synthetic fertilizers have increased from 548 MtCO_2_ in 2004 to 659 MtCO_2_ in 2014 and it was estimated that, in 2014, about 108 million tons of nitrogen fertilizers have been used worldwide and the 50% of it was used for cereal crops [15].

Emissions from synthetic fertilizers consist of direct and indirect N_2_O emissions from nitrogen added to agricultural soils by farmers. Direct N_2_O emission is produced by microbial processes of nitrification and denitrification taking place on the addition site. The indirect N_2_O emission is produced by: (i) a portion of volatilized ammonia (NH_3_) that will be deposited on soil and in water and be subjected to nitrification process and (ii) a portion of nitrate NO_3_^−^ that leaches and will be denitrified [16].

## 3. Plant Growth Promoting Bacteria: Plant Growth Promotion and Mechanism of Action

Soil is a complex system where the roots draw nutrients, ensuring the growth and development of the plants; on the other hand, the presence of insects, earthworms and microorganisms (fungi and bacteria) concur to influence the physiological and biological life of plants.

To ensure growth and productivity of crops, it is a common practice to add chemical fertilizers to quickly provide the essential nutrients to soil [17]. For the optimal growth and development of plants, herbicides, fungicides, insecticides are also added, but conversely, these compounds can damage plants and interfere with their ability to acquire some nutrients; an excessive use of these substances negatively affects human health and the environment [18],[19].

Therefore, the need to use alternative systems with a lower environmental impact to ensure the best growth of plants is increasing and the use of PGPB seems to be a valid proposal.

*Alcaligenes, Arthrobacter, Azospirillum, Azotobacter, Bacillus, Burkholderia, Enterobacter, Klebsiella, Pseudomonas* and *Serratia* are the most frequently microbial genera that have shown the ability to colonize plant rhizosphere and enhance plant growth [20].

Plants and microorganisms establish various relationships that facilitate beneficial (symbiotic and not) and/or harmful (pathogenic) interactions. During plant growth, microorganisms colonize the rhizosphere (defined by Walker et al. [21], as the narrow zone of soil directly surrounding the root system) and communicate with roots by producing plant growth-regulating substances. On the other hand, plants recognize microbe-derived compounds and modify their defense and growth mechanisms according to the type of microorganism [22].

These interactions occur both in phyllosphere (aerial plant surfaces) or rhizosphere (area surrounding the roots), but the environmental conditions for both sections are different. Phyllosphere is exposed to frequent changes of temperature, humidity, intensity light, etc., with consequent changes in nutrients availability. Concerning rhizosphere, it provides a better protection by changes in temperature and light intensity and guarantees a higher abundance of nutrients (more than 85% of the total organic carbon came from sloughed-off root cells and tissues or it is supply by plants as root exudates) [23]. Thus, numerous species of bacteria, fungi, protozoa and nematodes, colonize the rhizosphere, free or coated on the surface of the roots, by establishing very complex relations.

Concerning plant-microorganisms interactions, bacteria can act as free-living bacteria, symbiotic (that form a symbiotic relationship with roots), endophytes (that colonize only a portion of interior tissue of plant having a direct access to organic compounds), and cyanobacteria (formerly called blue-green algae) [24].

An example of the beneficial interaction between plants and microorganisms is the symbiotic interaction between the roots of legumes and some *Rhizobacteria* that lead to the formation of root nodules where the fixing of atmospheric nitrogen into ammonium, occurs [25]. *Rhizobium*, *Sinorhizobium*, *Bradyrhizobium*, *Mesorhizobium*, e *Azorhizobium* (generally known as Rhizobia) share the same habitat with PGPB and interact throughout the roots colonization. Lucas-Garcia et al. [26], Saharan and Nehra [20] and Bhattacharyya and Jha [27] in their studies confirmed the positive effects of this cooperation showing that PGPB can facilitate the nodulation process and improve the nitrogen-fixation in the roots of legumes.

Through direct or indirect mechanisms, or synergistically, PGPB can act on the enhancement of the performances of plants. Due to the direct action, PGPB provide the plants with some bacterial-synthesized compounds, modulate plant hormone levels that stimulate the proliferation of plant cells and facilitate the uptake of nutrients by fixing atmospheric oxygen, solubilizing minerals (such as phosphorus) and producing siderophores able to sequester iron. While, through indirect actions PGPB produce antagonistic substances or induce resistance against pathogens to prevent their harmful effects [28].

Saharan and Nehra [20], Glick [24], Bhattacharyya and Jha [27], Ahemad and Kibret [11], Beneduzi et al. [29] de Souza et al. [2], Kundan et al. [30] and Oteino et al. [31], published interesting reviews and articles on direct and indirect mechanisms; the most important findings are summarized in Table 1.

PGPB can be also employed as biocontrol agents, namely organisms able to kill other pathogens or organisms causing disease to crops. *Agrobacterium, Bacillus*, *Burkholderia, Pseudomonas* and *Streptomyces* belong to this group of PGPB [20]. The biocontrol activity of PGPB against soil-borne pathogens is due to mechanisms of microbial antagonism that results in a reduction of the saprophytic growth of pathogens and, consequently, in their frequency of infection. This reduction occurs through the competition for nutrients, colonization of habitats, induction of systemic resistance (ISR) in the plant host, production of antifungal metabolites. Furthermore, due to the ability of siderophore to sequester iron, PGPB subtract nutrients for the nutrition of pathogens [20].

*Pseudomonas* is the most important genus of *Rhizobacteria* acting as biocontrol agents; this is ubiquitous bacteria in agricultural soils. The way it uses to act as a biocontrol agent can be summarized as follows:

- It rapidly grows *in vitro*;
- It quickly uses seed and root exudates;
- It colonizes and multiplies in the rhizosphere and in the interior of the plant;
- It produces bioactive metabolites (i.e., antibiotics, siderophores, volatiles, and growth-promoting substances) and toxic metabolites (fenazine, pyrrolnitrin, 2,4-diacetylphloroglucinol (DAPG), pyoluteorin and cyclic lipopeptides [32];
- It competes with other microorganisms;
- It adapts to environmental stresses.

Unfortunately, pseudomonads are unable to produce resting spores (as do many *Bacillus* spp.) and this is a challenge to produce commercial formulates [20].

Among PGPB acting as biocontrol agents, a modified strain of non-pathogenic *Agrobacterium radiobacter* K84 has been used against *Agrobacterium tumefaciens*, responsible for the collar cancer. *A. radiobacter* K84 produces agrocina 84, an antibiotic compound, toxic for *A. tumefaciens*. Another application of *Agrobacterium* is due to its ability to survive and persist on the roots, which concurs to prevent the development of disease caused by pathogenic bacteria [33].

## 4. PGPB as Biofertilizers

Since 1990s some researchers have reported the potential of PGPB to promote plant growth and enhance the yield of crops in different soil and environment; in particular, *Azospirillum*, *Azotobacter*, *Bacillus* and *Pseudomonas* spp. were studied and applied in herbaceous crop systems as biofertilizers [34]–[38]. Some promising and controversial results on the mechanisms PGPB-plant root were reported [35],[36],[37],[39],[40]. There is not an official definition of biofertilizers; therefore, we refer to the definition of Vessey [41], “biofertilizer is a substance containing living microorganisms which, when applied to seed, plant surfaces, or soil, colonizes the rhizosphere or the interior of the plant and promotes growth by increasing the supply or availability of primary nutrients to the host plant”. This definition separates biofertilizers from organic fertilizers, which contain organic compounds and directly increase soil fertility. Therefore, biofertilizers should contain living organisms, which improve nutrient use efficiency of the host plant, through different mechanisms. A key factor, reported by Dobbelaere et al. [42], is the production of growth substances such as exogenous microbial auxins and gibberellins that showed stimulatory effect on plant growth. Vessey [41] also reported an estimated N contribution by PGPB N fixing prokaryotes of ca. 1–60 Kg/ha per year, thus the biofertilizers could reduce the need for chemical fertilizers and decrease adverse environmental effects.

Five mechanisms are mainly studied as modes of action of PGPB as biofertilizers: (1) biological N_2_ fixation, (2) increasing the availability of nutrients in the rhizosphere, (3) inducing increases in root surface area, (4) enhancing other beneficial symbioses of the host, and (5) combination of thereof [24],[41]. Table 2 offers an overview of the most important advances. To the best of our knowledge no study showed the application of “autochthonous bacteria” as biofertilizer. Moreover, the benefits of PGPB were observed only when organic or chemical fertilizers were added. A depth knowledge of (1) how and which bacteria are involved in N-transformation of “the modern” agricultural system and (2) abiotic factors (pH, temperature etc.) that control nitrification and denitrification is important to gain the application of PGPB in soil as biofertilizers.

**Table 1.** PGPB direct and indirect mechanisms.

DIRECT MECHANISMS	MICROORGANISMS
**PHOSPHATE SOLUBILIZATION** Some rhizosphere colonizing bacteria enhance phosphorous availability by making soluble, insoluble phosphate inorganic compounds or by liberating organic phosphates.	Bacteria: *Azotobacter*, *Bacillus*, *Beijerinckia*, *Burkholderia*, *Enterobacter*, *Erwinia*, *Flavobacterium*, *Microbacterium*, *Pseudomonas*, *Serratia* together with *Pantoea* and *Klebsiella, Rhodococcus*, *Arthrobacter*, *Chryseobacterium*, *Gordonia*, *Phyllobacterium* and *Delftia*. Rhizobia: *Mesorhizobium ciceri* and *Mesorhizobium mediterraneum.* Fungi: *Aspergillus* and *Penicillium*.
**NITROGEN FIXATION** Atmospheric nitrogen (N_2_) is not accessible to most living organisms but through Biological Nitrogen Fixation (BNF) it is reduced to ammonia (NH_3_). BNF is performed by symbiotic or non-symbiotic nitrogen fixing microorganisms.	Non-symbiotic: *Cyanobacteria*, *Acetobacter, Achromobacter, Alcaligenes*, *Anabaena*, *Arthrobacter*, *Azoarcus*, *Azomonas*, *Azospirillum*, *Azotobacter*, *Bacillus*, *Beijerinckia*, *Clostridium*, *Corynebacterium*, *Derxia*, *Enterobacter*, *Gluconoacetobacter diazotrophicus*, *Herbaspirillum* sp. *Klebsiella*, *Nostoc*, *Pseudomonas*, *Rhodospirillum*, *RhodoPseudomonas* and *Xanthobacter*. Symbiotic: *Rhizobia*, *Frankia*.
**INDOLEACETIC ACID (IAA)** IAA performs many functions: (i) affects cell division, extension and differentiation of plants, (ii) stimulates the germination of seeds and tubers, (iii) exerts a control on processes of vegetative growth, (iv) concurs to increase the rate of xylem and root growth, (v) initiates formations (lateral and adventitious) of the roots, (vi) modulates responses to light, gravity and fluorescence, (vii) affects photosynthesis, pigment formation, biosynthesis of various metabolites, (viii) resists to stressful conditions.	Several microbial species are able to produce IAA, through five biosyntetic pathways: (1) Saprophytic species of *Agrobacterium*, *Azospirillum*, some species of *Bradyrhizobium*, *Enterobacter*, *Erwinia herbicola*, *Klebsiella, Pseudomonas* and *Rhizobium* through via indole-3-pyruvic acid and indole-3-acetic aldehyde; (2) *Pseudomonads and Azospirilla* through the conversion of tryptophan into indole-3-acetic aldehyde with tryptamine as intermediate compound; (3) *Agrobacterium tumefaciens*, *E. herbicola*, *Pseudomonas fluorescens*, *Pseudomonas syringae* and *Pseudomonas putida* through the formation of indole-3-acetamide; (4) *Cyanobacterium* (*Synechocystis sp.*) through the formation of indole-3-acetonitrile; (5) *Azospirilla* and *Cyanobacteria* are also able to produce IAA through a tryptophan-independent pathway.
**SIDEROPHORE PRODUCTION** The production of siderophores can be classified as indirect mechanism when they are employed as means of biocontrol by microorganisms that do not use any other mechanism. By sequestering iron, PGPBs siderophore-producing reduce the availability of this element necessary for the growth of pathogens. Siderophore mediated iron scavenging in Gram-negative transport is better studied PGPB than Gram-positive PGPB.	*Bradyrhizobium japonicum*, *Rhizobium leguminosarum* and *Sinorhizobium meliloti* are the main microorganisms involved in siderophore production. Among Gram-negative bacteria, siderophore-producing belong to the *Pseudomonas* and *Enterobacter* genera, while *Bacillus* and *Rhodococcus* are the most representative among Gram-positive bacteria.
**ACC DEAMINASE ACTIVITY** 1-aminocyclopropane-1-carboxylate (ACC) deaminase is a bacterial enzyme involved to reduce the level of ethylene in plants. PGPB in response to tryptophan or other small molecules (see IAA section) synthetize and secrete IAA. This bacterial IAA, together with endogenous plant IAA, stimulate plant growth or induce the synthesis of the plant enzyme ACC synthase which converts the compound *S*-adenosyl methionine (SAM) to ACC, the immediate precursor of ethylene.	*Acinetobacter*, *Achromobacter*, *Agrobacterium*, *Alcaligenes*, *Azospirillum*, *Bacillus*, *Burkholderia*, *Enterobacter*, *Pseudomonas*, *Ralstonia*, *Serratia* and *Rhizobium* etc.
**PRODUCTION OF ANTIBIOTIC/ANTIFUNGAL METABOLITES** Phenazines, 2,4-diacetylphloroglucinol, pyoluteorin, pyrrolnitrin, lipopeptides, and hydrogen cyanide.	Fluorescent Pseudomonads, *Azospirillum*, *Azotobacter*, *Bacillus*, *Enterobacter*, *Paenibacillus*, and *Streptomyces*.
**PRODUCTION OF LYTIC ENZYMES** Chitinases, cellulases, β-1,3 glucanases, proteases, and lipases lyse a portion of the cell walls of many pathogenic fungi.	*Botrytis cinerea*, *Sclerotium rolfsii*, *Fusarium oxysporum, Phytophthora* spp., *Rhizoctonia solani*, and *Pythium ultimum*.
**COMPETITION FOR NUTRIENTS**	*Actinobacteria, Azospirillum brasilense, Bacillus amyloliquefaciens, Bacillus pumilus, Bacillus sp., Bacillus subtilis, Bacillus cereus, Bacillus licheniformis, Brevibacillus, Enterobacter sp., Jeotgalibacillus, Lysinibacillus, Paenibacillus, Paenibacillus polymyxa, Pseudomonas sp., Pseudomonas chlororaphis, P. fluorescens, Pseudomonas aeruginosa, Terribacillus*
**INDUCED SYSTEMIC RESISTANCE (ISR)** “Induced resistance” is referred to the induced state of resistance in plants triggered by inducers (biological or chemical); this state protects non-exposed parts against possible attack by pathogenic microbes and herbivorous insects.	*P. fluorescens*, *Serratia marcescens*, *Pseudomonas protegens*.

## 5. PGPB and Wheat with a Focus on Durum Wheat

Inoculation of PGPB to enhance performance and growth is particular interestingly in wheat. Veresoglou and Menexes [51] conducted a meta-analysis on 59 available articles focused on 228 field trials (only 12 trials on *T. turgidum* L. ssp. *durum*), and reported that *T. aestivum* ssp. *vulgare* may be a more responsive species when inoculated with *Azospirillum* spp. Moreover, the authors established linear regression models for the relationship between the effect sizes of seed yield and aboveground biomass separately for the field and pot trials. For durum wheat, the results must be confirmed. Some papers reported *Azospirillium* spp. and in particular *Az brasilense*, as one of the best PGPB able to promote growth, yield, nutrient uptake and productivity of wheat [47],[52]–[55].

**Table 2.** Application of biofertilizers in cereals.

Culture conditions	Species/*cultivar*	PGPB	Effects on plant growth and productivity	References
Field	*Triticum aestivum* L. cultivar Buck Suren^(R)^	*Azospirillum brasilense* and *Pseudomonas fluorescens*	Grain yield increases were not significant.	[35]
Field	*Triticum aestivum*	*Azospirillum* and *Azotobacter* sp	The inoculation increased: plant height, spike number per unit of area, grains number per spike, 1 000-grains weight, grain yield, biological yield and grain protein content.	[37]
*In vitro*	*Triticum aestivum* cv ProINTA	*Azospirillum brasilense* Sp245	The beneficial *Azospirillum*-wheat association is not hampered by the presence of Tebuconazole. *Azospirillum* increased root surface and promoted coleoptile length.	[43]
Field	*Triticum aestivum*	*Azospirillum brasilense*	The biofertilization reduced production costs and increased productivity.	[36]
Field	Wheat variety Zardana	*Azospirillum* and *Azotobacter* sp	The application of biofertilizer in combination with mineral fertilizer N 45 kg ha^−1^ and P_2_O_5_ 30 kg ha^−1^ increased fresh yield from 11% to 59% and grain yield by 20–46%.	[39]
Field	*Triticum aestivum*	*Methylobacterium* spp.	Increased plant growth and productivity, in an environment-friendly manner.	[44]
Field	Rice	*Pseudomonas fluorescens* and *Azospirillum brasilense*	PGPB inoculation increased aerial biomass production, harvest index, and grain yield of the Supremo 13 cultivar by 4.7%, 16%, and 20.2%, respectively.	[45]
Field	Corn	*Azospirillum* and *Azotobacter* sp	Reduction in production costs with increased productivity.	[38]
Greenhouse and field	Corn	*Azospirillum brasilense*	The inoculation of *Az brasilense* had the same grain yield when compared to nitrogen treatment. The grain production was increased by 29% in the treatment with *Az brasilense* and nitrogen compared to nitrogen fertilization alone.	[46]
Field	Durum wheat (cv. Anco Marzio)	*Bacillus* sp.	Soil inoculation with PGPB had a positive impact on plant growth in combination with organic fertilizer was added.	[40]
Field	*Triticum aestivum* L.	*Bacillus* sp. *Stenotrophomonas* spp. *Acetobacter pasteurianus* *Stenotrophomonas* spp	Plant growth-promoting *Rhizobacteria* (PGPR) provided a significant increase in shoot and root length, and shoot and root biomass. The study indicates the potential of these PGPR for enhancing growth and nutrient content of wheat and other crops under field conditions.	[47]
Pots and field	Wheat var. Inqlab-91	*Pseudomonas moraviensis* and *Bacillus cereus*	PGPR consortium with sugarcane husk and maize straw (biofertilizer formulation) increased growth, maintained osmotic balance and enhanced the activities of antioxidant enzymes and yield parameters.	[48]
Controlled conditions	Wheat	*Streptomyces* spp	These isolates can be used to design new biopesticides and biofertilizers with antibacterial and antifungal effect.	[49]
Field	Wheat, maize	*Paenibacillus polymyxa* WLY78	Nitrogen fixation, IAA production and phosphate solubilization performed by *P*. *polymyxa* WLY78 inside roots, stems and leaves and on root surfaces positively contributed to plant-growth promotion.	[50]

Pérez-Montano et al. [56] focused on the improvement of crop production by microorganisms for several cereals and leguminous and reported that in wheat, an ACC-deaminase producer *Pseudomonas fluorescens* strains concurred for the reduction of N, P and K fertilizer rates. Moreover, wheat crops resulted with higher grain yields, harvest index and protein content with lower fertilizer doses than those conventionally applied. An enhancement in grain yields was also found when two phosphate (PO_4_^3−^)-solubilizing microorganisms (PSM), *Bacillus circulans* and *Cladosporium herbarum* were combined with arbuscular mycorrhizal fungi (AMF). This kind of consortia affected also the grain and soil quality and the nutrient uptake of wheat.

There are many papers dealing with the benefits of PGPB towards legumes, maize, potatoes and wheat, but there are few reports on durum wheat. This is a crop cultivated in the Middle East, North Africa, the former USSR, the North American Great Plains, India, and Mediterranean Europe.

Durum wheat grows on 8 to 10% of all the wheat cultivated area [57]. Despite of its low acreage, durum wheat is economically important and is considered the hardiest of all wheats. Pasta is the excellent product derived from durum wheat but other products than pasta are also made from this cereal; couscous, made from durum semolina, is consumed mainly in North Africa; flat bread made from durum wheat and bulgur are consumed in Jordan, Lebanon, and Syria [57].

Depending on microorganisms, environmental and soil conditions, the interaction PGPB-durum wheat can act in different ways. Improvements in nutrient uptake (mainly N uptake), growth yield and grain quality, were reported by Saia et al. [40], Colla et al. [58], and Di Benedetto et al. [59].

Saia et al. [40] inoculated PGPB and arbuscular mycorrhizal fungi (AMF), alone and in combination, in durum wheat in a field experiment in Sicily. The authors observed that the presence of AMF in soil increased plant growth and N uptake of durum wheat compared to the uninoculated control irrespective of fertilization. PGPB provided beneficial effect on plant growth and nutrient uptake only when organic fertilizer was added. The authors concluded that soil inoculation with AMF and PGPB (alone or in combination) could be an alternative way for farmers to improve nutrient uptake and the sustainability of the agro- ecosystem, although further investigation are necessary.

Colla et al. [58] coated seeds of durum wheat with a microbial consortium of endophytic fungi (*Glomus intraradices* BEG72, *Glomus mossae* and *Trichoderma atroviride* MUCL 45632) with the aim to enhance growth, nutrient uptake, yield and grain quality. They found that this fungal cocktail enhanced the emergence time and shoot biomass of wheat seedlings, through an increase in root dry weight. Finally, an improved grain quality, in terms of protein, P, K and Fe concentration was recovered.

As few data are available on the interaction plant-PGPB isolated from Italian soil, in a recent work, Di Benedetto et al. [59] started a new research focused on the selection and characterization of PGPB, from a soil of South Italy (Capitanata, Apulia region) with high potential to enhance nutrient use efficiency. Competitive strains able to survive and establish in durum wheat rhizosphere, were isolated and three strains of *Pseudomonas* spp. showed characteristics of concern for the improvement of durum wheat nitrogen use efficiency.

Furthermore, two soils differing in both texture and organic carbon content, were sampled. At least about 400 isolates were collected. Odds in microbial cell number were observed in relation to the soil site. Mesophilic bacteria and actinobacteria showed the highest concentration. All the groups were tested in relation to the capacity to improve nitrogen availability and P-solubilization. In particular, some isolates of mesophilic bacteria, *Pseudomonas* spp., and actinobacteria were able to combine both nitrification and P-solubilization capacity. For the most promising strains a genetic characterization and a quantitative analysis of the parameters under study will be performed. Furthermore, the best strains will be inoculated in soil in order to test their ability to improve nutrient use efficiency in durum wheat.

Baffoni et al. [60] studied the interaction PGPB-durum wheat considering another point of view. They found that two bacterial strains, *Lactobacillus plantarum* SLG17 and *Bacillus amyloliquefaciens* FLN13 were able to reduce the incidence of Fusarium head blight (FHB), a severe disease caused by different *Fusarium* species. In a field experiment, a cocktail of the two microorganisms (applied from heading until anthesis) reduced FHB index and through some PCR-DGGE analyses the authors concluded that *L. plantarum* SLG17 was present in wheat seeds and probably act as endophytic bacteria. For these reasons, *L. plantarum* SLG17 and *B. amyloliquefaciens* FLN13 have been proposed as possible promising agents for the reduction of FHB incidence.

Mnasri et al. [61] studied the ability of sixty-two rhizospheric and endophytic bacterial strains *in vitro* and as seed coating for the control of two strains of *Fusarium culmorum* (Fc2 and Fc3) infecting durum wheat. The authors observed that 35% and 23% of the tested strains inhibited the *in vitro* growth of both strains. Some strains were able to produce volatile compounds that inhibit the growth, the sporulation and macroconidia germination of *F. culmorum*. The sequencing of the 16S rDNA genes of the bacteria showed that they belong to the genera *Bacillus*, *Pseudomonas* and *Microbacterium*. Then, *in vitro*, five strains were selected (four assigned to *Bacillus* and one to *Pseudomonas* genera) and inoculated together with two *F. culmorum*, in durum wheat. Results showed a reduction of the percentage of infected seeds and an improved germination and seedling vigor. Under greenhouse conditions, the virulence of the fungal strains and the specificity of the bacteria/fungi interaction, influenced the effectiveness of the biocontrol of *F. culmorum*.

## 6. A Focus on the Nitrogen Cycle and the Possible Role of PGPB

**Figure 2.**
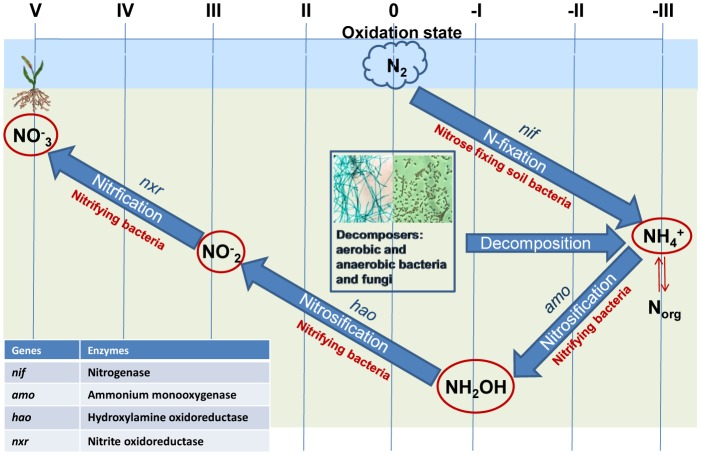
The major biological nitrogen pathways, that play a crucial role in transformation of fertilizing nitrogen in agricultural system (Some data were recovered by Canfield et al. [15]; the figure was an original work of the authors of this paper).

The core nitrogen cycle involves four reduction and two oxidation pathways. In particular, biotic Nitrogen Fixation (Figure 2) [15] involves *Azospirillum*, *Azotobacter* [24], *Pseudomonas*, *Acinetobacter* [62], *Klebsiella*, *Bradyrhizobium*, *Bacillus*, *Mesorhizobium*, *Rhizobium* [2],[11].

Ammonia oxidation is considered to be the rate-limiting step of nitrification [63] and has received greater scientific consideration than nitrite oxidation. It is catalyzed by an ammonia oxidizing bacteria (AOB, in particular *Nitrosomonas* sp.) and ammonia-oxidizing archaea (AOA) affiliated with *Thaumarchaeota phylum* [64].

The greenhouse gas N_2_O is a by-product of this process. AOB follow three distinct pathways: (i) nitrifier nitrification, (ii) nitrifier denitrification and (iii) nitrifier-coupled denitrification. During nitrifier nitrification N_2_O is formed as a byproduct of the spontaneous oxidation of hydroxylamine, instead nitrifier denitrification and nitrifier-coupled denitrification are stepwise reductions controlled by enzimes during which N_2_O is one of the intermediate that could escape in the atmosphere. It is not known why AOB perform nitrifier denitrification, one hypothesis is that it is a response to NO_2_^−^ toxicity under marginally aerobic conditions [65]. Alternatively, the coupling NH_4_^+^ oxidization to NO_2_^−^ reduction make nitrifier denitrification energetically favorable under marginally aerobic condition.

The last step is nitrite oxidation performed by NOB (e.g. *Nitrobacter*, *Nitrococcus*, *Nitrospina* and *Nitrospira*) [64],[66],[67]. Some studies highlighted a possible role of PGPB on both ammonium and nitrite oxidation [11],[24]. These hypothesis were confirmed by Di Benedetto et al. [59] who found some wild PGPB, putatively identified as *Pseudomonas* and *Bacillus* able to oxidize ammonia to NO_2_^−^ ions (nitrosification) and then to NO_3_^−^ ions (nitrification).

In nitrification processes the microbial functional importance changes depending on the kind of fertilization. In literature it is reported that in an alkaline soil the increase of nitrification by chemical nitrogen fertilizers is related to a change in community abundance and structure of AOB but not AOA [68]. As an effect of increased nitrogen fertilization a rising of AOB amo A genes and a little effect on AOA community composition [69] was observed.

The change of edaphic factors from excessive fertilization affects the great differences of nitrifiers in physiology and metabolic pathways [64]. In particular, Nicol et al. [70] considered the soil pH an important determinant of bacterial diversity and community structure because it probably influences the chemical form and availability of substrates. In fact, at low pH the growth and activity of ammonia oxidizers will be inhibited due to the increase of NH_4_^+^ ions despite of NH_3_ availability, the affinity to ammonia of AOB and AOA may drive to different growth [71].

Wang and Gu [67] reported that high salinity promoted bacteria growth but inhibits AOA; instead Erguder et al. [72] observed under low salinity (0.2–9 psu) and high C/N (12–25) that *archaeal amoA genes* were more abundant than *Betaproteobacteria amoA genes.*

In addition, the increases in organic matter favor AOA abundance and/or activity, while inorganic fertilizers lead to AOB and NOB dominated nitrification activity [66],[73].

## 7. N Uptake, N Assimilation and N Remobilization in Plants: a Focus on Wheat

N is the principal component of the proteins that build cell and plant tissue. Cereals and other plant species can utilize N as NO_3_^−^ and NH_4_^+^, which are the available inorganic forms of N absorbed by the roots from the soil solution [74]. In wheat, nitrogen is required to ensure photosynthetic activity, growth and grain yield and to produce grain storage proteins that have a key role in technological quality.

In wheat and rice, up to 80% of grain N content derives from leaves [75]. Most plants store nitrate in vacuoles and tolerate high ion concentrations, therefore nitrate might be also used an osmotic agent in plant [76]. Nitrate is used in various processes, including absorption, vacuole storage, xylem transport, reduction and incorporation into organic forms [77].

Nitrate assimilation is carried out mainly in the roots, being strongly dependent on the plant developmental stage and on the limitation of space for root growth [74]. In N assimilation process, nitrate is reduced to nitrite in the cytosol through the reaction catalyzed by the enzyme nitrate reductase (NR) using NAD(P)H as electron donors. The NR enzyme is positively regulated by NO_3_^−^ at light at the transcriptional level and is down-regulated at the post-transcriptional level by reversible phosphorylation during the dark period [77]. In hexaploid wheat, two genes encoding NADH-NR have been identified [78]. Since nitrite is highly reactive, plant cells immediately transport the nitrite from the cytosol into chloroplasts in leaves and plastids in root; in these organelles, nitrite is further reduced to NH_4_^+^ by nitrite reductase (NiR) [74]. NiR forms a complex with ferredoxin that provides electrons for the reduction of NO_2_^−^ to NH_4_^+^ [79].

Ammonia (NH_4_^+^) (inorganic N) is then assimilated into amino acids glutamine and glutamate, which serve to translocate organic N (N remobilization). The two main enzymes involved are glutamine synthetase (GS) and glutamate synthase or glutamine-2-oxoglutarate amino transferase (GOGAT), The GS is considered to be a possible rate-limiting step in ammonia assimilation. The synthesized amino acids glutamine and glutamate are used as amino group donors to all the other N-containing molecules, other amino acids used for storage, transport and protein synthesis and to nucleotides used as basic molecules for RNA and DNA synthesis [80].

## 8. Improvement of N-uptake Efficiency by the Interaction Root-PGPB

It is possible to identify three main characteristics/mechanisms of the rhizosphere that influence N-uptake efficiency (i) root size-morphology (ii) root N transporter system and (iii) interaction root-microorganisms such as PGPB.

It is reported that NO_3_^−^ and NH_4_^+^ uptake systems may be enhanced by the interaction with arbuscular mycorrhizal fungi (AMF) [8],[81], plant growth promoting bacteria (PGPB) [82], humic substances [83], allelopathic compounds such as cumarin [84] and inhibited by the increase of CO_2_ concentration in the atmosphere [85].

The use of plant growth promoting bacteria (PGPB) might be an alternative to increase NUE in important crops like wheat since these bacteria are able to increase root-system development and improve acquisition of nutrient including N.

NUE was evaluated through several methods (reviewed in Good et al. [86],[87]). According to an extensively used definition, plant nitrogen use efficiency (NUE) is defined as the grain yield produced per unit of applied N fertilizer. It is an integration of N uptake efficiency (NUpE) and N utilization efficiency (NUtE) defined respectively as the capacity of plants to acquire N from the soil and the fraction of plant-acquired N to be converted to total plant biomass or grain yield [8],[88],[89]. NUtE is very important to NUE of crops because its improvement would result directly in more biomass and yield.

Plant roots, including those of wheat, release organic acids, sugars, exudates and other rhizodeposits, which characterize the “rhizosphere”. Rhizodeposition can differ among wheat cultivars [90] leading to differences in various aspects of the rhizosphere microbial ecology [91]. In view of the increase of N-uptake it would be of interest to suppress pathogens and enhance root colonization by beneficial PGPB [8], in particular, those with the potential to enhance (a) N availability in the rhizosphere (N fixing bacteria and nitrifying bacteria), (b) root length and density (i.e IAA producer bacteria), (c) systemic plant metabolism and (d) microbial phytoprotection (i.e siderophores producer bacteria).

N availability is enhanced by microbial mineralization of organic N yielding ammonium in the rhizosphere (see Table 1). In wheat, the first effect is to attain higher N levels at flowering stage [92]. In particular, N availability for roots is improved by N fixation. Thus, the community of N fixers plays a key role for plant N nutrition [93]. In wheat and in other cereals, conversion of N_2_ into NH_3_ is performed by non-nodulating N-fixing bacteria. N-fixing bacteria occur naturally in soils including wheat rhizosphere [94],[95], and inoculation with N fixers may enhance wheat yield [40],[96],[97]. Their diversity and activity are influenced by plant species [98],[99] and cultivar [95],[100],[101],[102]. Several studies proposed the inoculation of *Azospirillium* spp. as N fixers bacteria [35],[37],[43],[52] to achieve higher yields.

Furthermore enhanced acquisition of water and mineral nutrients can be expected if the root system colonizes soil more extensively. Under *in vitro* conditions, wheat inoculation with rhizosphere bacteria may enhance root number and/or length, as well as root hair elongation [42],[103]. These inoculation effects on root system architecture and biomass have been also evidenced in wheat [51],[104]. These effects may be induced by the inoculation of PGPB producer of Indol Acetic Acid (IAA).

Many bacteria and fungi modify root system architecture by manipulating plant hormonal balance by producing phytohormones such as auxins [22], cytokinins [105],[106] or gibberellins.

For example, the wheat bacterium *Azospirillum brasilense* Sp245 synthesized abscisic acid, and modified lateral root development in *Arabidopsis* [107]. The effects appear to take place via auxin signal transduction pathway [8]. Microorganisms also interfere with ethylene metabolism in roots modifying wheat root development [108] by a direct microbial production of ethylene [109], or a reduction in ethylene concentration in plant roots by the deamination of ethylene precursor 1-aminocyclopropane carboxylic acid [110].

Some rhizosphere bacteria might directly affect N metabolism in plants. Oil seed rape (*Brassica napus* L.) roots inoculated with *Achromobacter* strain U80417 increased net influx rates of NO_3_^−^/NO_2_^−^ [111]. Furthermore, it is known that the inoculation of *Arabidopsis* with *Phyllobacterium brassicacearum* STM196 enhanced the coding of two nitrate transporters, NRT2.5 and NRT2.6 [112]. In wheat, nitrate reductase activity of *Azospirillum brasilense* Sp245 contributed to N assimilation [104]. PGPB might also improve N-uptake by promoting plant health by inhibiting root pathogens [24].

## 9. Conclusion

The use of PGPB could be a frontier goal to achieve a positive effect on plants and reduce the negative impact of chemical and fertilizers on the environment.

Some strategies have been tailored, but there are few reports on wheat. This review covers the actual knowledge with a focus on the gaps and on some possible future routes for the research. To better understand the interactions PGPB-plant, depth studies on the following issues are required: (i) the isolation of “autochthonous non-pathogenic PGPB” from rhizosphere in different environments; (ii) phenotypic and genotypic characterization to select PGPB strains able to enhance nitrogen use efficiency; (iii) the inoculation of selected PGPB under controlled conditions to study the interaction microorganisms-plant. These are necessary steps before the final application in field. This review offers a perspective on what the future could demand for.
